# Clathrin‐Mediated Endocytosis in Plants: Historical to Modern Advances

**DOI:** 10.1111/tra.70037

**Published:** 2026-05-15

**Authors:** Timber Mattson, Sebastian Bednarek, Alex Johnson

**Affiliations:** ^1^ Department of Biochemistry University of Wisconsin‐Madison Madison Wisconsin USA; ^2^ Biosciences University of Exeter Exeter UK

**Keywords:** actin‐independent, adaptor proteins, Arabidopsis, clathrin, dynamin‐related proteins, electron microscopy, plant clathrin‐mediated endocytosis, quantitative live cell imaging, TPLATE, vesicle proteomics

## Abstract

Clathrin‐mediated endocytosis (CME) is vital to plant physiology; however, the molecular details of how it functions in these complex multicellular eukaryotes remain to be defined relative to model yeast and animal systems. In this review, we explore potential reasons for this including a discussion of the debate of the existence of CME in plants, and the development of tools which have recently advanced our understanding of plant CME, including visualisation of live CME events in intact plants, and ultrastructural and proteomic analysis of plant clathrin‐coated vesicles. In addition, we present an updated schematic of the temporal and spatially distinct stages of plant CME that attempts to consolidate our current understanding and to serve as a working model for further study of plant CME. Plants occupy a distinct branch of the evolutionary tree of life relative to yeast and animal systems with their own cellular constraints (e.g., extremely high intracellular turgor pressure conditions). Thus, while the core CME machinery that was predicted to have already been present in the Last Eukaryotic Common Ancestor is conserved in plants, it has needed to evolve to overcome these plant cellular constraints. Thus, in this review, we wish to highlight plants as important model organisms for understanding the overall principles governing CME and how it can adapt in mechanically and biochemically distinct ways.

## Graphical abstract

Ultrastructural studies by Bonnett and Newcomb in 1966 revealed the existence of coated vesicles budding from the plant cell plasma membrane. This review introduces this discovery and early questions regarding the biological significance of CME in plants highlighting advances in our understanding of the plant CME molecular machinery along with current plant‑optimised experimental tools for its direct quantification.

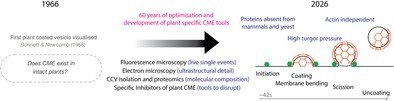

## 1 Introduction

Clathrin‐mediated endocytosis (CME), the major pathway for the internalisation of plasma membrane and extracellular materials, is essential for key plant growth and developmental processes including hormone signalling, nutrient uptake, and pathogen defence [1, 2, 3]. However, our understanding of the molecular machinery and dynamics of plant CME has lagged behind advances in the characterisation of CME in yeast and metazoans (Opisthokonts). This “cell biological” knowledge gap is surprising given that plants form the base of ecosystems, supply food and oxygen [4, 5, 6], and represent a major evolutionary branch distinct from Opisthokonts, offering unique insights into this evolutionarily conserved process. For example, the individual cells of plants are encased by rigid cell walls that result in high turgor pressure, often comparable to or exceeding those measured in other walled organisms such as yeast, as well as distinct plasma membrane sterol and lipid composition [7, 8]. In this review, we highlight work from the past two decades that has begun to precisely characterise the conserved and plant‐specific molecular mechanisms of CME.

Prior to these characterisations, many working models of plant CME were based largely on mechanistic assumptions derived from mammalian and yeast studies, owing to perceived cellular similarities, the presence of plant homologues of key endocytic proteins, and a reliance on indirect assays. However, the development of plant‐specific tools and optimised experimental approaches to examine CME at appropriate and multiple scales has led researchers to substantially revise our understanding and update our models, revealing that plant CME is mechanistically and evolutionarily distinct from well‐characterised mammalian and yeast systems.

**FIGURE 1:**
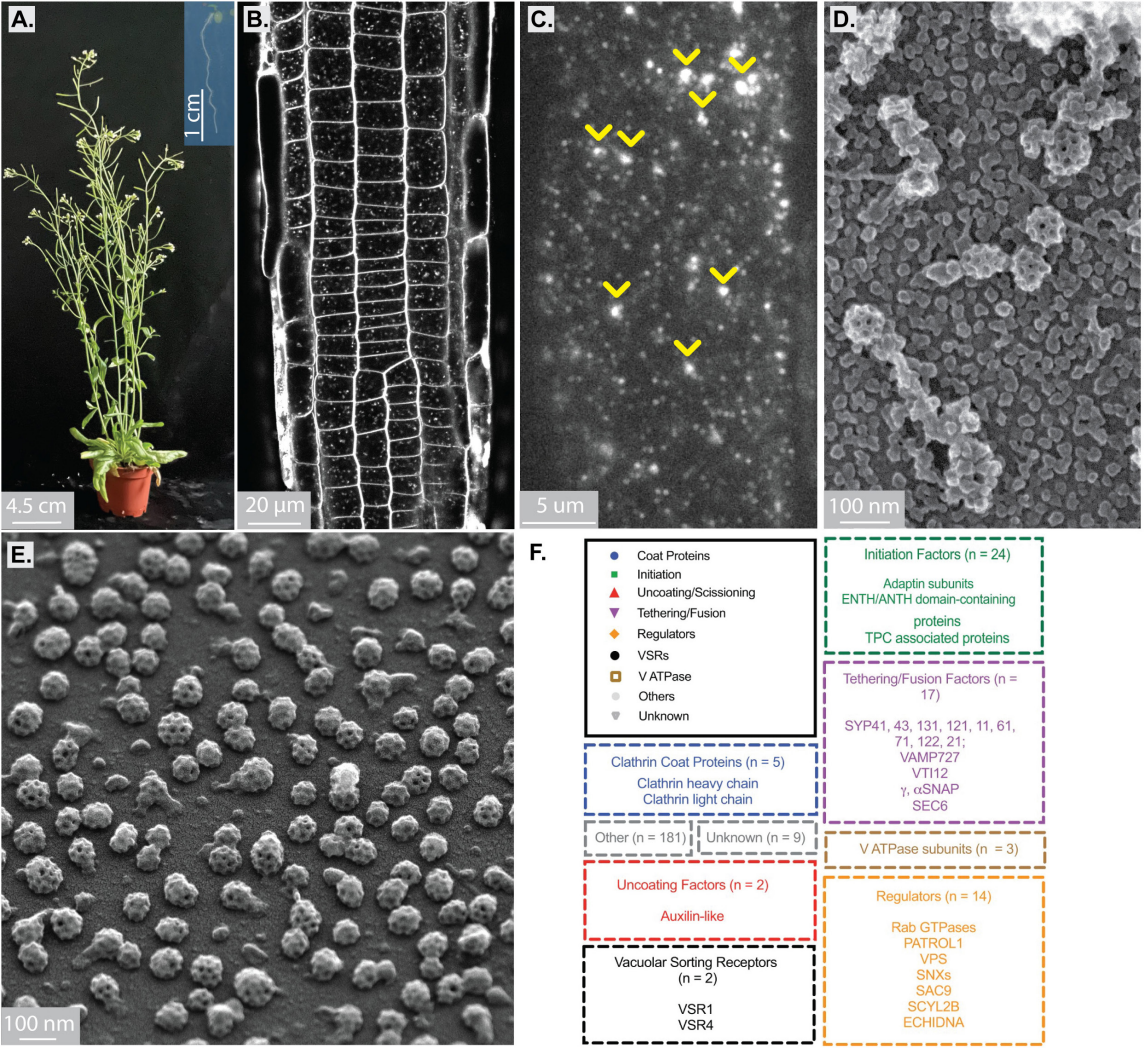
Plant clathrin‐mediated investigative tools from plant to protein scales. (A) A mature *Arabidopsis thaliana* plant grown on soil for ~6 weeks. The insert shows a 7‐day old seedling grown on agar, which is typically used for live imaging experiments when probing roots. (B) Example FM4‐64 uptake experiment conducted on an Arabidopsis seedling using a 40× objective on a Zeiss Airyscan 880. Image used with permission from [9]. (C) Example TIRF‐M image of an epidermal cell in a live and intact Arabidopsis seedling expressing CLC2‐TagRFP. The smaller spots represent single CME events, larger “blobs” represent actin‐linked mobile clusters of CCVs or pre‐endosomes (yellow arrowheads). Image taken from [10] which was published under a CC BY licence. (D) Example STEM image of a metal replicated unroofed Arabidopsis root protoplast. (E) Example STEM tilted image of isolated CCVs from Arabidopsis suspension cell cultures. (F) A list of enriched proteins found in plant CCVs identified using shotgun proteomics of isolated CCV preparations, as depicted in E. Image taken from [11] with permission. (D, E) are unpublished images taken by A. Johnson while supported by Prof. Jiri Friml at ISTA.

With recent advances in tools for studying clathrin‐dependent trafficking, plants have become a powerful biological model for CME, providing complementary perspectives on how fundamental eukaryotic cell biological processes operate in complex multicellular systems with distinct biophysical properties (e.g., high turgor pressure, rigid cell walls). It is also fitting to investigate the molecular mechanisms generating the clathrin “honeycomb lattice” in what Robert Hooke described as “honeycomb cells” in his seminal work *Micrographia*. This review explores the history and advances that have shaped our understanding of the molecular mechanisms of plant CME, focusing primarily on data from *Arabidopsis thaliana* (Figure 1A).

## 2 Discovery of Plant CME and Questions About Its Biological Significance

Coated vesicles (later defined as being encased by clathrin protein coat complexes) were visualised on the plasma membranes of radish root hairs in 1966 [12], only a few years after their identification by Roth and Porter in mosquito oocytes [13]. Subsequently, Barbara Pearse biochemically defined the observed vesicle coat as being clathrin, the triskelion protein complex comprised of clathrin heavy (CHC) and light (CLC) chains [14]. From the initial discovery of plant coated vesicles, it was another 20 years before clathrin‐coated vesicles were successfully isolated from plant cells [15, 16], and another 10 years until the clathrin heavy chain gene from soybean was cloned [17], confirming that clathrin is conserved in plants. Despite this, the existence and significance of CME in plants were questioned [18].

One reason for this debate stemmed from the fact that CME structures are rarely observed by electron microscopy in chemically fixed plant tissues. Thus, if one could not see endocytic structures at the plasma membrane of plant cells, then, by extension they likely didn't exist. However, CME structures had been readily visualised and examined in many plant cells and species; including *Allium cepa* L (onion), *Lepidium sativum* (garden cress), *Ceratopteris thalictroides* (water fern) and *Nicotiana tabacum* L (tobacco) [19, 20, 21]. Critically, these approaches made use of protoplasts—cells which have had their cell walls removed and thus have lower turgor pressure than intact plant tissues. Therefore, the debate evolved into “the turgor problem,” which proposed that while CME can function in protoplasts, it is energetically impossible for CME to overcome the high turgor pressure of intact plant cells (i.e., cells with their cell wall) (summarised by [22, 23]).

During this period of plant cell biology research, it was a challenge to directly test whether CME occurred in whole plant cells (non‐protoplasted) as the tools used in mammalian and yeast research were not yet optimised/available for plant samples (discussed in later sections), limiting researchers to the use of indirect approaches. For example, dyes that label membranes but cannot cross them, such as Lucifer Yellow CH (LY) and then later FM4‐64, were often used to demonstrate endocytic uptake in intact tissues [24, 25] (Figure 1B). However, LY uptake results were not always convincing; the dye was only found to be taken up in certain cell types [25, 26]. In contrast, FM dye labelling experiments demonstrated more reliable internalisation across a range of tissues and species [24, 27, 28]. Importantly, these dyes do not specifically label CME, but rather general and overall fluid‐phase or bulk plasma membrane uptake. While dye labelling studies showed that endocytosis was indeed functional in intact plant cells and tissues, it wasn't until later that the requirement of clathrin was confirmed. In 1996, clathrin was localised to the plasma membrane of pollen tubes by immunofluorescence microscopy [17]. Subsequently, the significance of clathrin in plants was shown through the expression of a dominant negative fragment of CHC, CHC‐Hub which corresponds to the C‐terminal trimerization domain of CHC [29]. In mammalian systems, overexpression of the CHC‐Hub domain acts as a dominant negative inhibitor of clathrin triskelion assembly, blocking CME [29]. Likewise, overexpression of the CHC hub domain in Arabidopsis protoplasts was found to disrupt clathrin coat formation, inhibit FM4‐64 uptake, and cause mislocalisation of plasma membrane endocytic cargo proteins (including PIN1, PIN2 and PIP2) [30] confirming that plant clathrin was indeed functionally significant for growth and development [30]. Critically, this was then utilised in transgenic seedlings, where the dominant‐negative CHC‐hub1 protein was shown to disrupt CME in roots and that CME is vital for plant development [31].

Thus, by developing direct and plant‐specific CME investigative tools, researchers demonstrated the existence and essential nature of CME for plant growth and development.

## 3 Current State‐of‐the‐Art Tools to Examine the Molecular Mechanisms of Plant CME

To uncover and probe the molecular mechanisms of plant CME, it is essential to examine it directly and at appropriate resolutions—where plant CME is < 42 s [32, 33], and requires precise spatial and temporal coordination of numerous proteins within an area of approximately 100 nm on the plasma membrane [34, 35, 36]. While tools to examine it at these scales and to specifically disrupt CME have existed in other model systems, significant optimisation was required to enable their use as well as the development of new tools for characterisation of plant CME, as plant cells present different and unique biological and technical challenges when compared to yeast and mammalian cultured cell systems.

### 3.1 Visualisation of Live Single CME Events in Intact Plants: Fluorescence Microscopy

Visualisation of single CME events by live cell imaging allows researchers to determine the localisation, dynamics, densities, and the temporal order of the recruitment of clathrin and its associated endocytic factors at the plasma membrane. Utilisation of such approaches for analysis of CME in intact plant samples has required extensive optimisation.

To capture CME events live, researchers have made use of fluorescence microscopy of plants expressing fluorescently labelled clathrin proteins (e.g., CLC2‐EGFP). Given the rapid nature of plant CME, spinning disk confocal and total internal reflection fluorescence microscopy (TIRF‐M) based approaches are best suited to investigate live CME events on the plasma membrane. TIRF‐M is favourable as it offers superior sensitivity and lower photon dosages, allowing more accurate and long‐term acquisitions. However, there was initial scepticism regarding the feasibility of TIRF‐M for imaging of the plasma membrane of intact plant samples. This stems from the fact that plants present several optical challenges: including a complex cell wall producing multiple refractive‐index mismatches, poor adhesion to coverslips, and an inherently “non‐flat” morphology. However, TIRF‐M is now well established for the investigation of plant CME and has been demonstrated to be compatible and effective for a range of intact plant tissues (e.g., [10, 37, 38]) (Figure 1C), it just typically requires different TIR angles from those optimised for mammalian cells. Because of this, early TIRF‐M investigations instead referred to TIRF‐M imaging as evanescent wave microscopy [39], variable‐angle epifluorescence microscopy (VAEM) [40], and more recently, variable‐angle TIRF‐M [38]. TIRF‐M has now become sufficiently routine that super‐resolution TIRF‐M approaches have been applied to intact plants; for example, TIRF‐SIM has been used to precisely define protein localisation at single CME events [41].

In parallel with these microscopy developments, CME quantitative analytic tools have also advanced greatly over the past decade. It is becoming routine to use high throughput and unbiased image analysis programs to measure CME dynamics, including TrackMate, uTrack and the detection and tracking of the cmeAnalysis package [9, 42, 43, 44], as opposed to manual measurements. Furthermore, researchers have developed a dual channel “departure assay” which defines the temporal profiles of proteins of interest at live single CME events from plasma membrane time lapses [9, 32, 45].

Thus, quantitative live cell fluorescence microscopy approaches allow the direct quantification of CME events and can define the precise temporal recruitment and spatial organisation of endocytosis proteins at the single event scale in intact plant cells.

### 3.2 Ultrastructural Visualisation of Plant CME Structures: Electron Microscopy

The high resolution of electron microscopy has facilitated the detailed characterisation of CME structures, including clathrin‐coated pit morphology and the precise organisation of the clathrin coat [46, 47, 48]. However, its use to probe the mechanisms of plant CME remains relatively limited.

As discussed above, many TEM sample preparation methods fail to robustly preserve CME structures in plant tissue sections. For example, to date, only a handful of publications have presented TEM images of CME in *Arabidopsis* tissues; including using chemical, high‐pressure freeze substitution and cryo fixation approaches (e.g., [49, 50, 51]). As an alternative approach, metal replication of plasma membrane fragments derived from “unroofed” protoplasts reliably preserves plant CME structures for EM investigation [9, 19, 20, 21, 32, 52] (Figure 1D). It is important to note that the plasma membrane‐associated CME structures derived from protoplasts have been formed under biophysical properties distinct from those of intact plant cells, critically where the turgor pressure has been removed. Nevertheless, their formation is dependent upon the same endocytic machinery as *in planta*. Indeed, the size of CME events found in both protoplasts and intact plant tissues does not significantly deviate [32, 53, 54], suggesting that their formation is similar.

To quantify plant CME events visualised by scanning electron microscopy (SEM) images of metal replicated unroofed cells, researchers have made use of manual measurements to quantify their diameters [9, 32]. More recently, deep learning and scanning transmission electron microscopy (STEM) tomography approaches have provided 3D morphological characterization of plasma membrane‐associated CME budding events and isolated clathrin‐coated vesicles (CCVs) [53].

Thus, while electron microscopy approaches for visualization of CME in intact plant tissues have remained challenging, metal replication of unroofed cells is a valuable alternative to directly and robustly quantify plant CME at ultrastructural resolutions.

### 3.3 Defining CCV Content: CCV Isolation and Proteomics

Methods for the subcellular fractionation and enrichment of CCVs, pioneered by Barbara Pearse and used for the identification of clathrin [14], coupled with mass spectrometry have provided significant insights into the identity of the proteins of interest involved in clathrin‐mediated trafficking (including CME) in mammalian cells [55, 56, 57].

By adapting the general methodology used by Pearse, plant CCVs were first isolated from suspension‐cultured carrot cells and pea cotyledons in 1986 and 1989, respectively [15, 16]. Isolation of CCVs in plant cells led to their initial morphological and biochemical characterization using negative stain electron microscopy and gel electrophoresis. However, a protocol for CCV isolation from Arabidopsis suspension‐cultured cells based off earlier plant CCV isolation was only established in 2014 [58] (Figure 1E) and for Arabidopsis seedlings in 2018 [54].

Proteomic analysis through isotope tagging or enrichment of Arabidopsis endomembrane compartment markers offered insights into the protein content of organelles such as the Golgi, trans Golgi network (TGN), early endosome (EE), late endosomes (LE), and multivesicular bodies (MVB) [59]. Likewise, a proteomic characterisation of CCVs enriched from actively dividing and expanding Arabidopsis suspension‐cultured cells was recently conducted [11] providing insights into the broad pool of CCV‐associated proteins and cargo, including coat proteins (clathrin heavy chains, clathrin light chains) and adaptor proteins (discussed in a later section) (Figure 1). Besides providing a vital starting point to uncover the mechanisms of endocytosis by identifying many novel target proteins of interest, it also further highlighted differences in CCV protein composition between plants and other models. Specifically, the adaptor protein AP4 was found to be associated with Arabidopsis CCV populations [11], which are not associated with CCVs in mammals or yeast. Additionally, affinity purification and proximity labelling of clathrin‐associated adaptor proteins further identified CCV interactors in Arabidopsis, including the plant homologue to mammalian p34, which has been suggested to function in maintaining adaptor protein populations to regulate CME and clathrin‐mediated post‐Golgi trafficking [60].

Together, isolation and biochemical examination of plant CCVs represent a powerful tool for proteomic studies of CCVs in plants to identify both conserved and divergent mechanisms between plants and other organisms. Future proteomic studies conducted on CME‐specific CCV populations could give more insight into endocytosis‐associated proteins and cargos that are involved in plant CME.

### 3.4 Specific Inhibitors of Plant CME

Pharmacological approaches are a common tool used to disrupt and thereby interrogate CME. However, many common inhibitors routinely used in yeast and mammals have weak or no effects on plant CME, highlighting the mechanistic divergence of plant CME from other model systems.

For example, Pitstop2 and Dyngo‐4a are reported to reduce, rather than inhibit, CME in plants [9, 61]. Furthermore, other well characterised inhibitors of mammalian CME act through alternative, off‐target mechanisms in plant cells. For example, Tyrphostin A23, which disrupts mammalian CME by targeting the AP2 complex [62], predominantly alters intracellular pH gradients in plant cells [63]. Researchers have also utilised Ikarugamycin to reliably block plant CME [9, 64], however, its mechanism of action remains unknown. To overcome these issues and provide a plant specific CME inhibitor, researchers have used chemical genomic approaches to screen for small molecules that target plant CME, including ES9‐17 that specifically inhibits clathrin heavy chain in both Arabidopsis and human cells [61].

**FIGURE 2:**
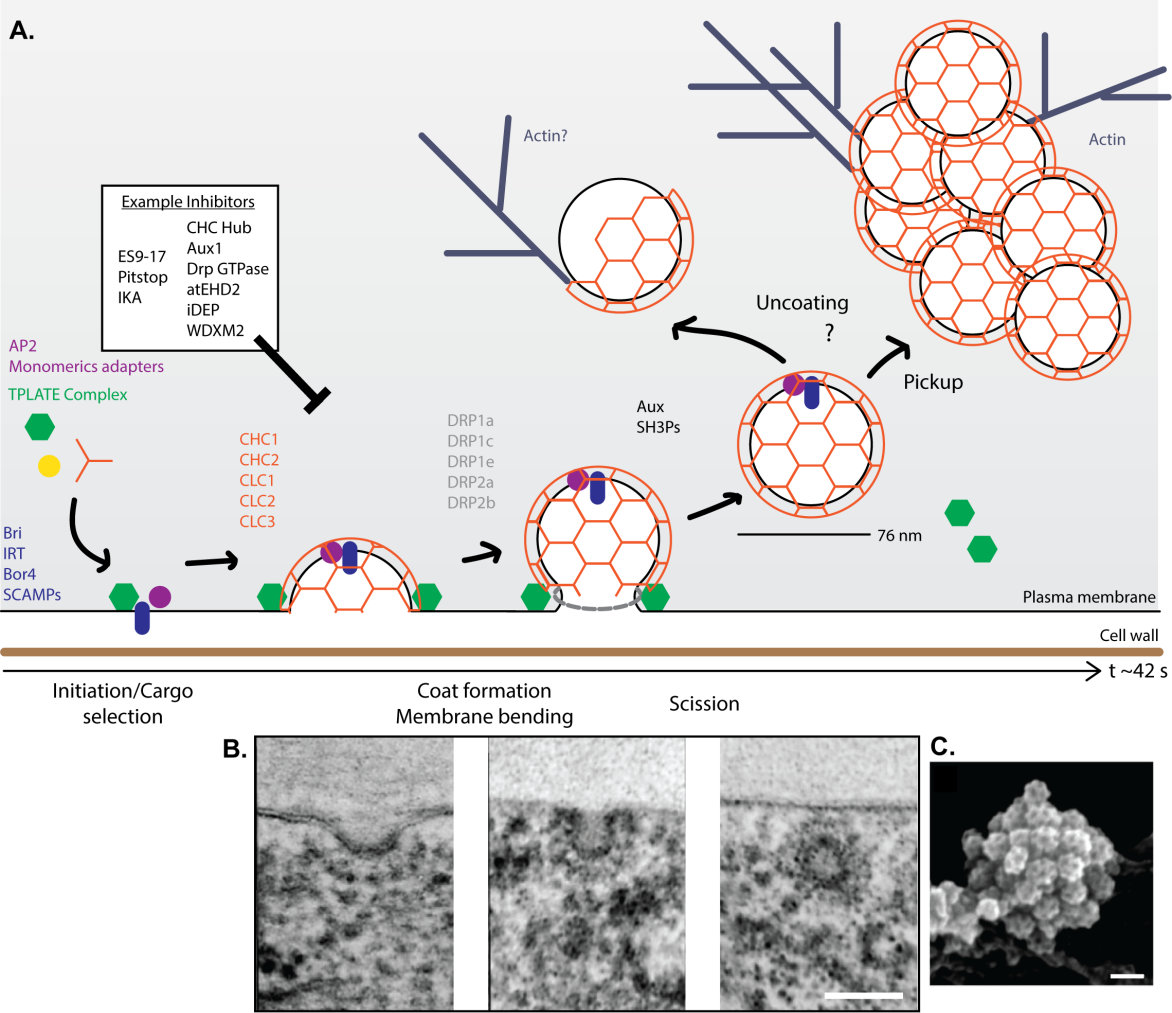
Arabidopsis plasma membrane clathrin‐mediated endocytosis. (A) Arabidopsis CME follows a stepwise progression; initiation/cargo selection, coat formation and membrane bending, scission and then uncoating. Initiation/cargo selection involves adaptor proteins (e.g., AP2 and monomeric adaptors [purple] and the TPLATE complex [green]) which bind the membrane, cargo (blue) and recruit other components of the endocytic machinery—like clathrin (orange). Plant CME appears to follow the “constant curvature” model, where the coat forms in concert with membrane bending, creating a dome and then omega shaped clathrin‐coated pit independently of actin. Scission of the clathrin‐coated membrane bud, potentially mediated by the plant DRPs (grey), releases the spherical CCV (diameter ~76 nm) from the plasma membrane. Endocytic CCV undergo actin‐mediated trafficking during which they display delayed or partial uncoating prior to fusion with the TGN/EE. In addition to this, the CCVs can aggregate in mobile clusters of CCVs (please see Figures 1C and 2C for example images of these structures). CME Initiation to scission in epidermal root cells takes 42 s. Inhibitors for plant CME are shown in the white box. (B) Example TEM images of the different stages of CME in Arabidopsis root protoplasts, taken from [30] with permission. Scale bar, 50 nm. (C) Example SEM image CCVs clusters in metal replicated unroofed Arabidopsis root protoplasts, taken from [32] which was published under a CC BY 4.0 licence.

Development of reverse genetic approaches for the inducible inhibition of plant CME has been enabled by having a complete annotated Arabidopsis genome, domain homology analyses and identification of critical plant endocytosis proteins. To date these tools include ones that target clathrin [30], the uncoating machinery [65, 66], and the initiation complex [67, 68] (Figure 2). However, to use them in conjunction with other fluorescently tagged proteins of interest requires a lengthy crossing of plant lines (~6 months).

Together, these challenges highlight the need for further development of plant‐specific tools and validation of pharmacological CME disruption approaches.

## 4 Molecular Mechanisms of Plant CME

### 4.1 Overview and Specific‐Plant Features

Plant CME follows a stepwise progression similar to the canonical model of CME developed from studies in metazoans and yeast (initiation and cargo selection, membrane bending and coating, scission), producing stereotypical structures (flat, dome and omega clathrin‐coated pits (CCPs)) at sites of endocytosis on the plasma membrane that resolve into spherical CCVs [30, 50, 51, 53] (Figure 2). In plant cells, this process is highly dynamic, where *bona fide* events are reported to have mean lifetimes of 42 s in intact Arabidopsis roots and 35 s in hypocotyls [32]. In addition to this population, which accounts for ~44% of clathrin on the plasma membrane, there are 2 shorter non‐productive populations of clathrin on the membrane [32]. Interestingly, in all the approaches used to examine CME in plants, there has been no evidence of the formation of clathrin plaques on the cell surface, supporting the idea that plant CME follows a canonical constant curvature mechanism [32].

One key difference between plant and mammalian and yeast CME is the presence of the TPLATE complex [68], known as the TSET complex in other non‐Opisthokont eukaryotes [69, 70]. The major difference between the TPLATE and TSET complexes is that the TPLATE complex is an octameric complex comprised of TPLATE, TML, LOLITA, atEH1/pan1, atEH2/pan1, TWD40‐1, TWD40‐2, and Tash3, whereas the TSET is hexameric—missing homologues to the atEH1/pan1 and atEH2/pan1 proteins [68, 69]. In plants, the TPLATE complex is essential for plant survival and CME [68], and while the precise functions of the complex remain to be fully understood, all of the individual subunits of the TPLATE complex contain domains that are homologous to those found in key mammalian and yeast endocytic proteins, the potential roles of which will be discussed further in the following sections.

A defining feature of plant CME is its ability to overcome the high turgor pressures found in plant cells. Turgor pressure can range from 1 to 4.5 MPa in stomatal guard cells (closed and open respectively) [71], ~0.1–0.4 MPa in pollen tubes, and ~0.3–1.5 MPa in root hairs, epidermal, mesophyll, parenchyma, and phloem cells [72]. The specific mechanisms by which the plant CME machinery can cope with these high levels of pressure and successfully generate CCVs, in under a minute, remain to be defined. However, unlike yeast CME which rely on actin‐based mechanisms to deal with high turgor pressure conditions, formation of plant endocytic CCVs appears to be actin independent [32, 33] (discussed in later sections).

### 4.2 Initiation

The initiation step of CME is perhaps the most difficult to characterise; however, the TPLATE complex is a good candidate to be a plant CME initiator. The TPLATE complex appears at CME events before clathrin [9], deforms membranes [41, 73], binds cargo [74, 75, 76] (Figure 2), and has conserved “initiation” domains found in mammalian and yeast endocytic proteins. Specifically, the TPLATE complex subunits contain Eps15 homology (EH) domains in atEH1/2 (homologue of mammalian Eps15 and yeast Ede1) and μ homology domain in TML (homologue of mammalian Fcho1 and yeast Syp1 [68]). These domains provide a crucial link between endocytic proteins and the membrane. The TML μ homology domain can bind directly to the membrane and links the atEH proteins to the TPLATE complex [76]. The EH domains can also directly interact with membrane lipids [76], and possess intrinsic membrane remodelling activity [41], enabling early deformation of the plasma membrane into clathrin‐coated pits. It has been suggested that phase separation of the atEH1 protein controls this initiation step [77], and similarly, this mechanism has also been suggested for mammals (Eps15) and yeasts (Ede1) [78, 79]. Interestingly, phosphorylation has been identified as a key regulator of phase separation [80], and atEH1 has been identified as a phospho substrate in response to environmental stress [81], suggesting a potential mechanism to activate plant CME in response to specific stimuli. However, it remains technically challenging to examine such potential phase separation mechanisms with native levels of proteins and at appropriate resolutions. It is also noteworthy that the role of the TPLATE complex in clathrin nucleation during CME is unclear. Overexpression and “knock‐sideways” (induced mis localisation to mitochondrial membranes [82]) of atEH1 results in clathrin assembly on mitochondrial membranes [77]. However, in cells carrying an inducible loss‐of‐function TPLATE mutant, which facilitates conditional disassembly/inactivation of the complex [67], clathrin lattices still assemble on the membrane [41] suggesting that additional factors are involved in clathrin recruitment during CME. For example, other proteins containing EH‐domains including atEHD1 and atEHD2 have been characterised as plant CME factors [83]. Interestingly, deletion of atEHD1 slowed endocytosis with no clear developmental phenotype, whereas overexpression of atEHD2 had an inhibitory effect on endocytosis [83].

Thus, while the precise mechanics of plant CME initiation remain unresolved, the TPLATE complex possesses homologues of key mammalian and yeast initiation domains (including EH and μ‐homology domains) and appears to be in the right place at the right time to fulfil this role.

### 4.3 Cargo Adaptors

The major role for CME in plant growth and development is the internalisation and subsequent degradation or recycling of proteins from the plasma membrane. However, while plants possess homologues to multimeric and monomeric adaptors, the mechanism for cargo selection in plants is not entirely clear.

*Adaptor protein 2 (AP2)*: Plants possess the AP2 complex (Arabidopsis has Alpha, beta, mu and sigma subunits), that interact with clathrin in vivo and were found associated with CCVs in proteomic studies [11, 84]. Arabidopsis mutants lacking AP2 subunits are viable but display severe growth defects [85]. Underlying these phenotypes is the fact that AP2 has been linked to the internalisation and mediation of physiologically significant receptors and transporters; including BRI1, IRT1, BOR1, PIN2, cellulose synthase complex proteins, CESA3 and CESA6 [74, 86, 87, 88, 89]. It has also been demonstrated that plant AP2 also makes use of canonical AP2‐cargo recognition motifs; specifically, YxxØ in BRI1 [87], IRT1 [88], and PIN2 [86]. Interestingly AP2‐dependent endocytosis of BOR1 is not mediated by this motif [89]. Furthermore, in another divergence from mammalian AP2 function, plant AP2 is reported to function similar to *C. elegans* AP2 as two hemi‐complexes [90, 91].

*TPLATE complex*: The TPLATE complex has been reported to drive cargo selection during CME, including internalisation of the receptor kinase CLAVATA1 which is required for shoot apical meristem maintenance [92]. Furthermore, additional TPLATE complex subunits have been shown to be required for the CME‐dependent internalization of specific cargos. Specifically, TWD40‐2 and TML were found to function in CME of the CESA cellulose synthase complex in a manner distinct from AP2 [74, 93], whereas atEH1/Pan1 mediates Secretory Carrier Membrane Protein 5 (SCAMP5) endocytosis via binding and recognition of a double N‐terminal NPF motif within SCAMP5 [76]. In addition, the SH3 domain of TASH3 has been implicated in the internalisation of ubiquitinated cargos [75]. Interestingly, relative to the central core of a CCP on the plasma membrane, the TPLATE complex appears to be spatially positioned on the exterior side of these structures, and proteomic analysis shows only low abundance of the TPLATE complex with budded CCVs [11, 41], displaying distinct temporal dynamics at CME events compared to AP2 [68, 74]. Thus, while the TPLATE complex possesses classic adaptor function by directly linking cargo to the clathrin coat, the mechanisms by which it selects and transfers cargo inside the clathrin‐coated pit (CCP) remain to be defined.

*Monomeric adaptors*: Plants possess monomeric adaptors containing AP180‐N‐terminal‐homology (ANTH) and Epsin N‐Terminal homology (ENTH) domains that facilitate interactions with membrane lipids (PI(4,5)P_2_), adaptor complexes (AP2 and TPLATE) and clathrin [94, 95] to promote clathrin assembly and regulate CCV size [96]. While ANTH proteins are largely conserved across all eukaryotes, in Arabidopsis there are 18 ANTH proteins compared to the smaller families found in mammals and yeast [97]. Of these, the main CME‐associated ANTH proteins in plants are AP180/PICALM, EPSIN‐LIKE CLATHRIN ADAPTOR1 (ECA1)/PICALM1a, ECA2/PICALM5a, ECA4/PICALM4a, and CLATHRIN‐ASSOCIATED PROTEIN1 (CAP1)/PICALM4b all of which have been found to be associated with CCVs in Arabidopsis [2, 11]. These plant ANTH proteins are also conserved in non‐vascular land plants (i.e., bryophytes) [97, 98]. Interestingly however, an additional kinase domain‐containing ANTH designated PI‐CALM‐K was identified in the liverwort *Marchantia polymorpha* that is not present in moss *Physcomitrium patens* [98] or Arabidopsis.

In addition to ANTH type monomeric adapters, seven ENTH proteins are present in Arabidopsis, three of which have been identified as CCV interactors in proteomic analyses: EPSIN1, EPSIN2, and modified transport to the vacuole 1 (MTV1) [11]. EPSIN1 and MTV1 are both localized to the TGN and associate with AP1 and AP4, respectively, and are not thought to play a role in CME [99]. In contrast, AtEPSIN2 is implicated in CME and binds to AP2 and the SNARE VTI12 [100]. Interestingly, AtEPSIN2 also interacts with the adaptor protein complex AP3, which appears to not be associated with plant CCVs [11], indicating that EPSIN2 may also play a role in clathrin‐independent trafficking [100]. Besides ANTH and ENTH domains, there is also a family of monomeric adaptors containing VPS27, HRS, STAM1/STAM2 (VHS) domains; one subgroup is the TOM1‐Like (TOL) family. There are 9 members in plants, where TOL6 and TOL9 are localised to the plasma membrane and are thought to recognize ubiquitinated cargo and recruit for degradation through the ESCRT pathway [101, 102]. Although their function in either CME or clathrin‐independent endocytosis is still undefined, both TOL6 and TOL9 possess putative clathrin‐binding domains and were found to associate with the TPLATE subunit atEH1 and CCVs through proteomic analyses [77].

Therefore, while plants possess functional homologues to canonical eukaryotic adaptors, there are divergences in their molecular mechanism. Furthermore, the TPLATE complex represents a further divergence, where its additional domains relative to the TSET complex appear to also have vital cargo adaptor roles.

### 4.4 Clathrin Coat Assembly

The core of the coat, and a key ingredient for CME, is repeating units of clathrin assemblies. Clathrin is postulated to have been present in the last eukaryotic common ancestor (LECA) [103], and in 2015 it was reported that clathrin has been found in the genome of all but one sequenced eukaryote [104]. Consistent with this, plants have both clathrin‐heavy chains (CHCs) and clathrin light chains (CLCs).

In Arabidopsis, there are two CHC genes (atCHC1 and atCHC2) sharing 91% sequence identity and 73% with human CHCs [34]. They also have strong domain homology and organisation to human CHCs, suggesting they assemble to form triskelia—the base unit of the clathrin coat—in a similar way. Specifically, the C‐terminal central hub of CHC binds to other CHCs, while the N‐terminal leg extensions are involved in binding to CLC and adaptor proteins to mediate triskelia multimerization [105]. Indeed, ultrastructural studies of plant CME events and isolated CCVs have shown that clathrin triskelia combine to form the stereotypical honeycomb hexagon and pentagon arrangements of the clathrin coat (Figures 1D,E and 2). Interestingly, the two atCHCs isoforms encoded in the Arabidopsis genome appear to be expressed together in the same cells [31], and although it has been reported that loss of CHC2 results in more severe growth defects than CHC1, the isoforms do not appear to be fully redundant with each other [31, 106]. Arabidopsis has three genes for CLCs (atCLC1, atCLC2 and atCLC3), and while atCLC2 and atCLC3 share 60% sequence similarity, atCLC1 displays more divergence with only about 50% similarity with the other isoforms [34]. Loss of function *clc1* mutants display a pollen lethal phenotype [107], whereas loss of function *clc2* and *clc3* mutants have viable pollen but display pleiotropic defects in plant development that increase in severity in the *clc2clc3* double mutant [107]. Compared to human and yeast CLCs, all atCLCs have an extra C‐terminal disordered domain, whose function remains to be determined [34]. Furthermore, while mammalian CLCs possess a highly conserved domain at their N‐terminus involved in binding the ANTH proteins HIP1 and HIP1R to regulate CHC multimerization and triskelia formation [108, 109, 110], this domain is not conserved in plants. Interestingly, it was found that co‐localisation of CHC1 and CLC2 was only ~60% on the plasma membrane [32], suggesting specificity of interactions between the CHCs and CLCs.

Plant clathrin has also been investigated in the green algae *Chara australis*. Here, clathrin has been implicated in CME to facilitate the dismantling of charasomes, which are convoluted PM domains involved in carbon uptake for photosynthesis [64]. CaCHC1/2 share 75% identity with each other and are similar to land plant CHCs. In addition, *C. australis* encodes two CLC genes (CLC1/2), each with distinct splicing variants. These CLC genes are more divergent than the heavy chains, with CaCLC1a–c and CaCLC2a,b sharing 26%–93% sequence identity, suggesting the possibility of functional and cell‐specific differences comparable to mammalian CLCs.

While the plant CHCs are heavily conserved and produce a stereotypical clathrin coat pattern, the plant CLCs appear more diverse. Furthermore, it is not clear what the functional consequences of having multiple clathrin isoforms in the same cells are and if there are specific populations of CCVs made from a variety of combinations of CHC and CLCs.

### 4.5 Membrane Bending

Membrane bending is essential during CME as it remodels the flat plasma membrane into an invaginated CCP, which is internalised as a CCV (Figure 2). Based on the mechanistic principles derived from mammals and yeast, in which actin is required to drive membrane bending under conditions of high turgor pressure and membrane tension [111], it had long been assumed that actin must be required for plant endocytosis as well due to the very high turgor pressure in plant cells. However, by systematically examining live single events of CME in multiple *Arabidopsis* tissues with actin perturbations, researchers found no changes in CME dynamics or densities, indicating that plant CME occurs independently of actin [32, 33]. Instead, actin is vital in post‐endocytic trafficking (Figure 2), as actin disruptions resulted in mis‐localisation of receptors [32]. This actin‐independence of plant CME has also been demonstrated in other plant models; for example, actin inhibition has no effect upon FM dye uptake in *Chara* [27]. Together, this indicates that plants rely upon an alternative mechanistic solution to overcome turgor.

One potential solution to overcome this is the role of the TPLATE complex [41]. Indeed, live cell and ultrastructural examination of plant CME events following conditional depletion of the TPLATE protein revealed that the TPLATE complex is essential for endocytic membrane bending [41]. While TPLATE complex members possess intrinsic membrane bending activity in vitro and in structural simulations [41, 73], it is not clear if the TPLATE complex is responsible for driving membrane bending directly, or if it is critical in recruiting other unknown membrane bending proteins, or both.

Together, these findings indicate that plants have evolved mechanisms to drive membrane bending against high turgor pressure distinct from other model systems, but how exactly it is achieved remains elusive.

### 4.6 Scission

To create the CCV, the CCP must be cut/freed, or “scissioned,” from the plasma membrane. In mammals, this process is typically mediated via dynamin; a GTPase with a C‐terminal effector domain, a pleckstrin homology domain (PH) that aids in membrane binding, and a proline‐rich domain (PRD) to aid in protein–protein interactions with SH3‐containing proteins [111]. Thus, members of the plant dynamin‐related protein (DRP) 2 protein family, which contain the same domains and organisation, are strong candidates to be the plant CME scission machinery.

The DRP2 family contains two members, DRP2A and DRP2B, which share 93% sequence identity and are thought to be functionally redundant [112, 113]. They have been found to interact with CCVs and locate at CME events [114, 115], and dominant negative GTPase versions blocked endocytic marker uptake and resulted in bursting of expanding root hairs [116], together highlighting a critical role in plant CME and plant growth. However, direct evidence that they function in scission of CCVs is still lacking. Interestingly, the temporal behaviour of the DRP2s does not mirror that of mammalian dynamin, which has a sharp burst of its bulk recruitment seconds prior to CCV scission [117]. Instead, the DRP2s show a gradual recruitment throughout the whole CME reaction [45, 114], suggesting a potential mechanistic divergence. Furthermore, recruitment of the DRP2 proteins to CME events was thought to be mediated via its proline‐rich domain (PRD), specifically by binding to SH3P2, a protein with an SH3 domain and a BAR domain homologous to endophilin and amphiphysin—proteins known to recruit dynamin to mammalian CME events [111]. However, more recent work has shown that SH3P2 does not directly recruit DRP2 to CCPs [45].

Arabidopsis possess another DRP family which has been implicated in CME, the DRP1 family. It has 5 members (DRP1A‐E) which lack the PH and PRD domains found in dynamin and the atDRP2s. While several members of the DRP1 family display functional redundancy, DRP1c mutants are lethal [118]. Their involvement in CME has been determined by co‐localisation of DRP1A and DRP1C with CLC2 and DRP2 on the plasma membrane [33, 114, 119] as well as dominant negative GTPase null DRP1A and knockdowns of DRP1A/C/E that blocked and disrupted endocytic cargo uptake and recycling, respectively [119, 120]. However, in vitro reconstitution assays found that while DRP1A can bind liposomes, it cannot cut/scission them [121].

DRP2 and DRP1 likely have synergistic functions in CME. *drp1a drp2b* double mutants exhibit severe defects in growth and development and display defects in ligand‐induced endocytosis of the plant pattern recognition receptor FLAGELLIN SENSING2 (FLS2) involved in innate immune defenses, indicating synergistic roles for DRP1A and DRP2B in the internalisation of FLS2 [122]. Furthermore, the temporal recruitment of DRP1A to CME events appears to be gradual throughout the CME reaction [123], like DRP2. Proteomic analysis of Arabidopsis CCV fractions showed only low associations of DRP2 and DRP1A/C with CCVs [11], indicating that they are indeed likely to be localised outside the CCP to fulfil a potential scission role.

While the DRPs are likely candidates to be the plant scission machinery, direct evidence for their involvement in this step remains to be demonstrated. There also appears to be a divergence in the temporal behaviour of the DRPs compared to the mammalian dynamins, highlighting further potential mechanistic differences of endocytic membrane remodelling in plants.

### 4.7 Uncoating

To process cargos encapsulated in CCVs, the coat needs to be removed. However, little has been uncovered about the uncoating mechanisms of CME CCVs in plants. Arabidopsis possesses homologues to canonical uncoating proteins; notably AUXILIN‐LIKE1 and 2, which contain DNAJ domains, and HSP70s [65, 124]. Overexpression of AUXILIN‐LIKE1 prevents CME, significantly reducing the concentration of clathrin on the plasma membrane, but not AP2 and TPLATE—suggesting that its overexpression can disrupt clathrin recruitment and/or availability [65, 66]. Furthermore, it has been recently reported that a BAR domain containing protein family (SH3Ps) could recruit AUXILIN‐LIKE1 to sites of CME [125], similar to the role of endophilin (a BAR domain containing protein) which is required for efficient uncoating in mammals [126]. Binding of AUXILIN‐LIKE1 to CCVs is hypothesized to function in the recruitment of chaperone proteins, like HSP/HSCs. Interestingly, deletion of HSP70‐9 was found to result in a decrease of plasma membrane clathrin, and in vitro analysis found HSP70 dissociates clathrin from isolated CCV preparations [124]. However, live CME imaging found that only ~5% of CME events had AUXILIN‐LIKE1 recruited to them, and its deletion produced no observable phenotypes [65], suggesting additional factors are required for the uncoating of CCVs.

Resolving these uncoating mechanisms has been further complicated by the fact that plant CME CCVs do not undergo uncoating as promptly as those in yeast and mammals. Imaging experiments revealed that the uncoating process does not occur immediately after scission and that CCVs form “clusters” [32] (Figure 2). These CCV clusters associate with actin filaments and appear as large, highly mobile structures when imaged by TIRF‐M, which have been postulated to represent an early endosome structure—however, these structures have lacked a detailed characterisation. Interestingly, there appears to be a coordination between these large CCV structures and the termination of CME [32], where the newly generated CCVs appear to be “picked up” by the mobile structure. Furthermore, when plant cells are exposed to osmotic stress, it has been reported that CCVs can be targeted directly to autophagosomes [49], highlighting an alternative route whereby CCVs may bypass uncoating altogether.

Together, these observations indicate that CCV uncoating in plants occurs later in the trafficking pathway than in other systems, and that the molecular regulation and functional consequences of delayed uncoating remain poorly understood.

## 5 Issues and Future Challenges

One of the biggest challenges in understanding plant CME is that to date the majority of studies have been conducted in Arabidopsis. Thus it remains to be determined whether the principles learned in this one model system are conserved across the diversity of plants. Other developing model plant systems such as the extant representatives of early land plants *Marchantia polymorpha* and *Physcomitrium patens* afford the ability to rapidly conduct reverse genetic studies and to integrate fluorescent fusion proteins into candidate genes via homologous recombination, thus avoiding overexpression artifacts [127, 128]. These techniques, not yet possible in Arabidopsis, will likely contribute to the understanding of plant CME and its evolution.

This “Arabidopsis focus” limitation is further compounded by the fact that Arabidopsis CME studies are largely restricted to live cell imaging of CME events in the epidermal cells of the root or hypocotyl, particularly on the accessible lateral edge or in tip‐growing root hairs and pollen tubes [33, 94]. As such, this working model of plant clathrin‐dependent trafficking is based upon plasma membrane CME. Interestingly, clathrin‐dependent trafficking from the Golgi appears to function without the presence of TPLATE and the DRPs and remains to be characterised [129].

One reason other CME proteins have not been examined to the same extent is that plants are not readily amenable to transient transfection protocols, so researchers must generate new genetically edited plants—which requires substantial time and commitment (typically ~6 months per line). Furthermore, one key tool missing from plant CME research is a *bona fide* model cargo analogous to transferrin in mammalian studies. Although many cargos have been identified, those that are endocytosed constitutively or when triggered by ligands are difficult to work with; for example, IRT1 requires specific growth conditions for expression [130] and BRI1 internalises through multiple pathways [87, 131]. This search for a model receptor is so critical that researchers have even expressed human transferrin in plant cells [132].

In addition to characterising plant CME proteins, the role of lipids in this process requires further investigation. Lipids play an essential role in CME in yeast and metazoans [111]. Similarly, studies in plants have indicated that they are critical for endocytosis (reviewed in [35]). In particular, the recent development of the rapid and reversible inducible depletion of phosphatidylinositol 4,5‐bisphosphate (PI(4,5)P2) system in Arabidopsis has demonstrated the critical requirement of PI(4,5)P_2_ in plant CME [133]. But because of a lack of plant‐specific tools that provide sufficient spatial and temporal resolution for CME events, we know little about their function in plant endocytosis. However, the mammalian field has recently developed novel mammalian lipid probes, so perhaps with some plant‐specific optimisations we can overcome these limitations [134, 135].

While plant CME characterisation has enjoyed a renaissance being driven by advances in tools specifically designed to probe plant CME at direct and high resolutions, there is still much to discover. It will be interesting to see how future insights into its mechanistically and evolutionary distinct mechanisms apply to our overall fundamental understanding of eukaryotic cell biology.

## Funding

A.J. is supported by a University of Exeter research fellowship from the faculty of Health and Life Science. Open Access funding provided by The University of Exeter. S.B. and T.M. are supported by funding from the National Science Foundation (MCB‐2439581, MCB‐2154572).

## Conflicts of Interest

The authors declare no conflicts of interest.

## Data Availability

The authors have nothing to report.
